# The comments of voices on the appearance of patients with psychosis: ‘the voices tell me that I am ugly’

**DOI:** 10.1192/bjo.2019.66

**Published:** 2019-09-20

**Authors:** Felicity Waite, Rowan Diamond, Nicola Collett, Eleanor Chadwick, Emily Bold, Ashley-Louise Teale, Kathryn M. Taylor, Miriam Kirkham, Eve Twivy, Chiara Causier, Lydia Carr, Jessica C. Bird, Emma Černis, Louise Isham, Daniel Freeman

**Affiliations:** Research Clinical Psychologist, Department of Psychiatry, University of Oxford; and Oxford Health NHS Foundation Trust, UK; Research Assistant, Department of Psychiatry, University of Oxford, UK; Research Assistant, Department of Psychiatry, University of Oxford and Oxford Health NHS Foundation Trust, UK; Professor of Clinical Psychology, Department of Psychiatry, University of Oxford; and Oxford Health NHS Foundation Trust, UK

**Keywords:** Schizophrenia, hallucinations, obesity, weight, body image

## Abstract

**Background:**

There are high rates of obesity and low self-esteem in patients with psychosis. The occurrence of negative voice content directly about appearance is therefore plausible. Derogatory comments about appearance are likely to be distressing, increase depression and contribute to social withdrawal.

**Aims:**

To systematically assess the occurrence of voice content regarding appearance and identify correlates.

**Method:**

Sixty patients experiencing verbal auditory hallucinations at least once a week in the context of non-affective psychosis completed a measure assessing positive and negative voice content about appearance. They also completed assessments about body image, self-esteem, psychiatric symptoms and well-being.

**Results:**

Fifty-five (91.7%) participants reported hearing voices comment on their appearance. A total of 54 (90%) patients reported negative voice content about their appearance with 30 (50%) patients experienced negative appearance comments on a daily basis. The most common negative comment was ‘the voices tell me that I am ugly’ (*n* = 48, 80%). There were 39 (65%) patients who reported positive voice content on appearance. The most frequent positive comment was ‘I look as nice as other people’ (*n* = 26, 43.3%). Negative voice content about appearance was associated with body image concerns, paranoia, voice hearing severity, depression, worry, negative self-beliefs and safety-seeking behaviours. Positive appearance voice content was associated with greater body esteem and well-being and lower levels of depression and insomnia.

**Conclusions:**

Voice content about appearance is very common for patients seen in clinical services. Negative voice content may reflect – and subsequently reinforce – negative beliefs about one's appearance, low self-esteem, worry and paranoia.

**Declaration of interest:**

None.

Excess weight is a major problem for many patients with psychosis. More than 50% of patients have a body mass index (BMI) in the obese range^1^ – this is double the rate for adults in the general population.^2^ Potential causes include the adverse effects of antipsychotic medication, genetic vulnerabilities, metabolic syndrome, psychosocial risk factors and unhealthy lifestyles.^3,4^ There is a widely recognised period of rapid weight gain following antipsychotic initiation.^3^ Excess weight contributes towards lower levels of both physical health and psychological well-being. For example, significant weight gain is associated with low self-esteem, social isolation, poor quality of life and non-adherence to medication.^5^ Depression, low self-esteem and social withdrawal resulting from excess weight and associated concerns about appearance may exacerbate psychotic experiences.^6^ Indeed, concerns about body weight have been shown to be associated with paranoia.^7^ A recent qualitative investigation with patients with psychosis indicates that body image concerns extend beyond weight to include dissatisfaction with facial features, hair, clothing and overall physical attractiveness.^6^ Notably, patients also described how such appearance-related concerns can be a source of vulnerability that connects to both paranoia and voices.^6^

Given the high rates of obesity^1^ and low self-esteem in patients with psychosis,^8^ the occurrence of negative voice content about appearance or body image is plausible. Yet, to date, there have been no quantitative investigations of voice content regarding appearance. In this study we set out to systematically assess voice content regarding appearance in patients currently hearing voices in the context of non-affective psychosis. The primary question was whether voices comment about appearance. We also tested whether the voice content relates to weight, self-perceived body image, self-esteem, worry, depression and paranoia. In addition, we tested whether voice content about appearance was associated with difficulties sleeping, activity levels and psychological well-being. Candidate variables were identified based on patient accounts,^6^ theoretical models^9^ or having been established as common clinical problems in patients with psychosis.^10^ Our expectation was that hearing voices talk about one's appearance may have associations with multiple psychological, psychiatric and functioning domains.

## Method

### Participants

The participants were 60 patients who reported hearing voices at least once a week, as assessed on the Cardiff Anomalous Perceptions Scale (CAPS) Hallucinations subscale.^11^ The patients were recruited as part of The Feeling Safe clinical trial (trial registration: ISRCTN18705064)^12^ from three National Health Service (NHS) mental health trusts: Oxford Health NHS Foundation Trust, Berkshire Healthcare NHS Foundation Trust and Northamptonshire Healthcare NHS Foundation Trust. Ethical approval was received from an NHS Research Ethics Committee (South Central – Oxford 78 B Research Ethics Committee; ref 15/SC/0508). Written informed consent was received from all participants.

The trial evaluates a novel psychological intervention for persistent persecutory beliefs. The inclusion criteria for the trial were as follows: a current, persistent (at least 3 months) persecutory delusion (as defined by Freeman & Garety, 2000),^13^ held with at least 60% conviction; a primary diagnosis of schizophrenia spectrum psychosis (non-affective psychosis); aged 16 years or above; and willing and able to give informed consent for participation in the trial. The exclusion criteria were: current receipt of another psychological therapy; insufficient comprehension of English; primary diagnosis of alcohol/drug misuse or personality disorder; receiving treatment in a forensic service; organic syndrome; or intellectual disability. Patients hearing voices at least weekly completed an assessment of voices commenting on appearance during the baseline assessment for the trial.

### Measures

Basic demographic and clinical data were collected (for example age, gender, ethnicity, employment status, clinical diagnosis).

#### Weight and body image

##### Body-Esteem Scale for Adolescents and Adults (BESAA)

The BESAA is a 23-item self-report scale assessing self-evaluation of appearance.^14^ Each item (for example ‘I like what I look like in pictures’) is rated on a 0 (never) to 5 (always) scale. There are three subscales: ‘appearance’ includes general feelings regarding appearance (10 items) (for example ‘I like what I see when I look in the mirror’, ‘I feel ashamed of how I look’), ‘attribution’ includes evaluations attributed to others about one's appearance (5 items) (for example ‘Other people consider me good looking’), and ‘weight’ relates to satisfaction with weight (eight items) (for example ‘I am satisfied with my weight’, ‘My weight makes me unhappy’). Higher scores indicate greater levels of positive body esteem.

##### BMI

This is a value derived from an individual's weight and height. It is calculated by dividing an individual's weight in kilograms by their height in meters. BMI can be used to categorise an individual as underweight (<18.5), normal weight (18.5–24.99), overweight (25–29.99) or obese (>30). The obese range has subcategories: class I (30–34.99), class II (35–39.99) and severe or morbid (>40).

#### Psychotic experiences

##### Voices Commenting on Appearance (VOCA)

This 20-item self-report measure was developed specifically for this study (the items can be seen in Table 1). The measure was developed in collaboration with a patient advisory group. Item content was produced drawing upon patient accounts, the authors' clinical experience and existing scales measuring relevant concepts, for example the BESAA.^14^ The measure includes items in which: (a) voices directly comment on an individual's appearance (weight, general appearance, clothes, attractiveness), and (b) voices comment on others' evaluation of the individual's appearance. Domains include: weight, clothing, general appearance, sexual attractiveness, shame, social evaluation and perceived difference from others. Each item is rated on a 5-item scale: 0 (never), 1 (rarely), 2 (once a month), 3 (once a week), 4 (several times a week) or 5 (daily). The measure includes negative (*n* = 12) and positive (*n* = 8) items.

**Table 1:** Mean scores on the measures

Variable	*n*	Mean	s.d.
Body esteem (BESAA)	59	28.77	16.81
BESAA, appearance subscale	59	14.18	8.47
BESAA, attribution subscale	59	5.57	4.04
BESAA, weight subscale	59	9.02	6.57
Body mass index	56	30.82	7.05
Voices and appearance, negative	60	28.44	17.10
Voices and appearance, positive	60	5.16	6.66
Hallucinations (CAPS)	60	19.20	4.73
Paranoia (GPTS)	60	114.37	23.17
Negative self-beliefs (BCSS)	60	12.98	5.19
Positive self-beliefs (BCSS)	60	7.35	4.59
Depression (BDI)	60	33.25	10.70
Worry (PSWQ)	60	62.82	10.56
Insomnia (ISI)	60	14.15	7.10
Safety-seeking behaviours (SBQ)	60	35.52	15.37
Avoidance (SBQ)	60	19.13	9.45
In-situation defence behaviours (SBQ)	60	16.38	8.87
Well-being (WEMWBS)	60	33.30	8.11
Activity (Time budget)	60	55.75	13.14

BESSA, Body Esteem Scale for Adolescents and Adults; CAPS, Cardiff Anomalous Perceptions Scale; GPTS, Green et al Paranoid Thoughts Scale; BCSS, Brief Core Schema Scale; BDI, Beck Depression Inventory; PSWQ, Penn State Worry Questionnaire; ISI, Insomnia Severity Index; SBQ, Safety Behaviours Questionnaire; WEMWBS, Warwick-Edinburgh Mental Wellbeing Scale.

The maximum possible score for the negative items is 60. Higher scores indicate more frequent and prevalent negative comments from voices regarding appearance. The maximum possible score for the positive items is 40. Higher scores indicate more frequent and prevalent positive comments from voices regarding appearance. Both subscales demonstrated acceptable internal consistency (negative items Cronbach's α = 0.93, positive items Cronbach's α = 0.88).

##### Cardiff Anomalous Perceptions Scale (CAPS) hallucinations subscale

Five items assessing voice hearing were used from the scale.^11^ Each item (for example ‘Hear voices commenting on what you're thinking or doing’, ‘Hear noise or sounds when there is nothing about to explain them’, ‘Hear two or more unexplained voices talking to each other’) is rated on a 0 (not at all) to 5 (daily) scale. Higher scores indicate greater levels of hallucinatory experiences.

##### Green et al Paranoid Thoughts Scale (GPTS)

This 32-item scale measures paranoid thinking.^15^ Part A assesses ideas of reference (for example ‘It was hard to stop thinking about people talking about me behind my back’) and part B assesses ideas of persecution (for example ‘I was convinced there was a conspiracy against me’). Each item is rated on a 5-point scale. Higher scores indicate greater levels of paranoid thinking.

#### Negative affect and related processes

##### Brief Core Schema Scales (BCSS)

The BCSS comprises 24 items assessing negative and positive beliefs about the self and others, each rated on a five-point scale (0–4).^16^ Four subscale scores are obtained: negative self (for example ‘I am unloved’), positive self (for example ‘I am respected’), negative other (for example ‘Other people are hostile’), positive other (for example ‘Other people are fair’). Higher scores indicate greater endorsement of items.

##### Beck Depression Inventory-II (BDI)

The BDI-II is a self-report 21-item scale, with each item rated on a 4-point scale (0–3), for the assessment of depression over the past fortnight.^17^ Higher scores indicate higher levels of depression.

##### Penn State Worry Questionnaire (PSWQ)

This 16-item scale measures trait worry.^18^ Each item is rated on a 5-point scale. Higher scores indicate a greater tendency to worry.

##### Insomnia Severity Index (ISI)

The ISI is a seven-item self-report questionnaire assessing insomnia symptoms over the past fortnight.^19^ Each item is rated on a 0–4 scale. Higher scores indicate the presence of symptoms of insomnia.

##### Safety Behaviours Questionnaire (SBQ) – persecutory delusions

The SBQ is a semi-structured interview assessing safety behaviours used in the past month.^20^ An action is deemed a safety behaviour if the interviewee reports that it has been carried out with the intention of reducing persecutory threat. A distinction is made between avoidance of situations and in-situation safety behaviours (for example not making eye contact). The number and frequency of safety behaviours are calculated to produce a total score. Frequency is rated on a 4-point scale from 1 (behaviour definitely occurred on at least one occasion) to 4 (present more or less continuously/at least every day). Higher scores indicate a greater number and frequency of safety behaviours.

##### Psychological well-being

The Warwick-Edinburgh Mental Well-being Scale (WEMWBS)^21^ is a 14-item scale assessing well-being over the past fortnight. Each item is rated on a 1 (none of the time) to 5 (all of the time) scale, and therefore the total score can range from 14 to 70, with higher scores indicating a greater level of well-being.

##### Activity levels

The Time budget^22^ measure assesses meaningful activity levels over the past week, completed during a structured interview, with four time blocks for each day rated from 0 to 4. The rating scale is: 0, nothing; 1, predominantly passive activity; 2, an independent activity requiring some planning and motivation; 3, several 2-rated activities completely filling a time period or a more complex and demanding, but shorter, activity; 4, time period filled with a variety of demanding independent activities. Higher scores indicate higher levels of meaningful activity.

### Analyses

This was predominately a descriptive study, with the main reporting involving the proportion of the participants experiencing appearance-related voice content and frequency of endorsement of specific voice content. Associations between voice content and the symptom and psychological variables were tested using Pearson correlations. To test differences between BMI categories an ANOVA was conducted. All statistical testing was two-tailed and carried out with SPSS Version 22.0.

## Results

### Demographic and clinical information

The average age of the participants was 41.9 (s.d. = 11.7) years old. There were slightly more men (*n* = 36) than women (*n* = 24). The ethnicities were: White (*n* = 49), Black Caribbean (*n* = 4), Indian (*n* = 3), Pakistani (*n* = 2), Black African (*n* = 1) and other (*n* = 1). Most participants were single (*n* = 42), with others married or in a civil partnership (*n* = 12), cohabiting (*n* = 1) or divorced (*n* = 5). The majority were unemployed (*n* = 54). Most patients had a BMI in the obese range (*n* = 28). No patients had a BMI in the underweight range. The clinical diagnoses were schizophrenia (*n* = 37), schizoaffective disorder (*n* = 13) and psychosis not otherwise specified (*n* = 10). All but one of the patients (*n* = 59) were currently prescribed antipsychotic medication. Most participants were out-patients (*n* = 57) at the time of participation. Table 1 reports the mean scores for all clinical measures.

### Prevalence and frequency of voice content regarding appearance

Voice content regarding appearance was highly prevalent: 55 (91.7%) patients reported that voices have made comments about their appearance and 53 (88.3%) patients reported hearing voices commenting on their appearance on at least a weekly basis. There were 32 (53.3%) patients who reported that voices made comments about their appearance on a daily basis. Table 2 summarises the endorsement of the individual items on the VOCA.

**Table 2:** Endorsement of individual voice content about appearance items

The voice(s) tell me…	Never, *n* (%)	Rarely, *n* (%)	Once a month, *n* (%)	Once a week, *n* (%)	Several times a week, *n* (%)	Daily, *n* (%)
I am ugly	12 (20)	9 (15)	5 (8.3)	5 (8.3)	14 (23.3)	15 (25)
People don't like me because of how I look	13 (21.7)	9 (15)	3 (5)	5 (8.3)	15 (25)	15 (25)
My body is disgusting	14 (23.3)	6 (10)	6 (10)	9 (15)	10 (16.7)	15 (25)
People think I am ugly	14 (23.3)	8 (13.3)	2 (3.3)	8 (13.3)	15 (25)	13 (21.7)
I look worse than others	14 (23.3)	13 (21.7)	6 (10)	4 (6.7)	12 (20)	10 (16.7)
I should be ashamed of my appearance	15 (25)	10 (16.7)	7 (11.7)	5 (8.3)	12 (20)	11 (18.3)
No-one will fancy me	15 (25)	9 (15)	6 (10)	5 (8.3)	9 (15)	16 (26.7)
I look crazy	15 (25)	8 (13.3)	5 (8.3)	9 (15)	10 (16.7)	13 (21.7)
People think I am fat	16 (26.7)	3 (5)	4 (6.7)	6 (10)	13 (21.7)	18 (30)
My clothes are embarrassing	17 (28.3)	12 (20)	7 (11.7)	8 (13.3)	7 (11.7)	8 (13.3)
I am fat	19 (31.7)	7 (11.7)	3 (5)	4 (6.7)	11 (18.3)	15 (20)
I am too skinny	44 (73.3)	9 (15)	2 (3.3)	2 (3.3)	2 (3.3)	1 (1.7)
I look as nice as other people	34 (56.7)	17 (28.3)	0 (0)	4 (6.7)	4 (6.7)	1 (1.7)
My clothes are stylish	36 (60)	16 (26.7)	0 (0)	4 (6.7)	2 (3.3)	2 (3.3)
I am good looking	36 (60)	14 (23.3)	4 (6.7)	4 (6.7)	1 (1.7)	0 (0)
I have a nice figure	39 (65)	12 (20)	4 (6.7)	3 (5)	1 (1.7)	0 (0)
People fancy me	40 (66.7)	14 (23.3)	1 (1.7)	2 (3.3)	1 (1.7)	2 (3.3)
I look like everyone else	40 (66.7)	11 (18.3)	3 (5)	3 (5)	1.7 (1)	2 (3.3)
People think I am good looking	42 (70)	10 (16.7)	3 (5)	4 (6.7)	1 (1.7)	0 (0)
I should be proud of how I look	43 (71.7)	10 (16.7)	1 (1.7)	3 (5)	2 (3.3)	1 (1.7)

#### Negative voice content about appearance

Fifty-four patients (90%) reported that voices made negative comments about their appearance. Only six patients (10%) reported that voices have never made negative comments about their appearance. Negative comments about appearance were frequent: 52 (86.7%) patients reported hearing negative comments about their appearance on at least a weekly basis and 30 (50%) on a daily basis. The total mean score for negative comments was 28.4 (s.d. = 17.1). Scores ranged from 0 to 55 (potential maximum 60). It can be seen in Table 2 that the most frequently endorsed negative comment was ‘the voices tell me I am ugly’ (*n* = 48, 80%). The most frequently endorsed negative comment occurring on (at least) a daily basis was ‘the voice(s) tell me people think I am fat’ (*n* = 18, 30%). However, only 15 (20%) patients reported daily comments from voices commenting directly on their weight: ‘the voice(s) tell me I am fat’. The least frequently endorsed negative comment was ‘I am too skinny’ (*n* = 16, 26.7%).

#### Positive voice content about appearance

Thirty-nine (65%) patients reported that voices made positive comments about their appearance. Twenty-one (35%) patients reported that voices have never made positive comments about their appearance. Positive voice content about appearance occurred on a weekly basis for 18 (30%) patients and on a daily basis for just five (8.3%) patients. The total mean score for positive comments was 5.2 (s.d. = 6.7). Scores ranged from 0 to 30 (potential maximum 40). The most frequently endorsed positive item was, ‘I look as nice as other people’ (*n* = 26, 43.3%), followed by, ‘my clothes are stylish’ (*n* = 24, 40%) and ‘I am good looking’ (*n* = 24, 40%). However, these comments were predominantly rated as occurring ‘rarely’ (*n* = 17, 28.3%; *n* = 16, 26.7%, and *n* = 14, 23.3% respectively). The least frequently endorsed positive comments were, ‘I should be proud of how I look’ (*n* = 17, 28.3%) and ‘People think I am good looking’ (*n* = 18, 30%).

### Correlates of voice content

Table 3 reports the correlations between voice content about appearance and the symptom variables and psychological constructs. Negative voice content regarding appearance had a medium negative association with body esteem. Conversely, positive voice content regarding appearance had a medium positive effect size associated with body esteem. There were significant associations in all three subscales of the BESAA: appearance, attribution and weight. For example, there was a large effect size association between the attribution scale and positive voice content regarding appearance.

**Table 3:** Correlates of appearance-related voice content

	Total negative voice content	Total positive voice content
Body esteem (BESAA – appearance subscale)	–0.449*** P < 0.001	0.490*** P < 0.001
Body esteem (BESAA – attribution subscale)	–0.302* P = 0.020	0.558*** P < 0.001
Body esteem (BESAA – weight subscale)	–0.374** P = 0.004	0.422** P = 0.001
Hallucinations (CAPS)	0.476*** P < 0.001	–0.033 *P* = 0.804
Paranoia (GPTS – total)	0.373** P = 0.003	0.028 *P* = 0.830
Negative self-beliefs (BCSS – negative self)	0.473*** P < 0.001	–0.384** P = 0.002
Positive self-beliefs (BCSS – positive self)	–0.321* P = 0.012	0.262* P = 0.043
Depression (BDI)	0.326* P = 0.011	–0.481*** P < 0.001
Worry (PSWQ)	0.277* P = 0.032	–0.194 *P* = 0.137
Insomnia (ISI)	0.056 *P* = 0.672	–0.441** P < 0.001
Defence behaviours (SBQ – total)	0.366** P = 0.004	–0.150 *P* = 0.251
Avoidance (SBQ)	0.251 *P* = 0.053	–0.217 *P* = 0.97
In-situation defences (SBQ)	0.367** P = 0.004	–0.030 *P* = 0.820
Wellbeing (WEMWBS)	–0.203 *P* = 0.121	0.456** P < 0.001
Activity (time budget)	0.096 *P* = 0.465	0.016 *P* = 0.901

BESSA, Body Esteem Scale for Adolescents and Adults; CAPS, Cardiff Anomalous Perceptions Scale; GPTS, Green Paranoid Thoughts Scale; BCSS, Brief Core Schema Scale; BDI, Beck Depression Inventory; PSWQ, Penn State Worry Questionnaire; ISI, Insomnia Severity Index; SBQ, Safety Behaviours Questionnaire; WEMWBS, Warwick-Edinburgh Mental Wellbeing Scale.

*P < 0.05, **P < 0.01, ***P < 0.001.

There were significant differences between BMI categories in the endorsement of negative voice content (*F*(4,51) = 3.719, *P* = 0.010), but not for positive voice content (*F*(4,51) = 1.021, *P* = 0.405). Table 4 reports the mean scores on the VOCA by BMI category. BMI was significantly positively associated with specific voice comments, for example ‘people think I am fat’ (*r* = 0.421, *P* = 0.001) and negatively correlated with ‘I am too skinny’ (*r* = −0.354, *P* = 0.007).

**Table 4:** Mean scores on the Voices Commenting on Appearance measure by body mass index category

	*n*	Mean	s.d.
Positive voice content about appearance			
Normal weight	14	6.48	8.23
Overweight	14	5.43	5.67
Obese, I	13	7.23	8.70
Obese, II	10	1.90	2.23
Obese, morbid	5	4.20	5.36
Total	56	5.37	6.82
Negative voice content about appearance			
Normal weight	14	20.03	15.01
Overweight	14	30.57	16.68
Obese, I	13	22.92	15.34
Obese, II	10	42.67	16.39
Obese, morbid	5	23.80	9.88
Total	56	27.71	16.91

Total voice hearing severity, measured on the CAPS, hallucination subscale, had a medium effect size association with negative voice content but no association (*r*<0.1) with positive voice content. There was a medium effect size correlation between negative voice content and paranoia. There was no association between paranoia and positive voice content regarding appearance (*r*<0.1).

Negative voice content regarding appearance was positively associated with negative beliefs about the self (medium effect size) and negatively associated with positive beliefs about the self (medium effect size). Conversely, positive voice content regarding appearance was positively associated with positive beliefs (small effect size) about the self and negatively with negative beliefs about the self (medium effect size). There was a small but non-significant correlation between negative and positive voice content about appearance (*r* = −0.108, *P* = 0.410). In contrast, there was a medium effect size negative correlation (*r* = −0.409, *P* = 0.001) between negative and positive beliefs about the self.

Negative voice content regarding appearance was positively associated with depression (medium effect size), conversely, positive voice content had a medium effect size negative association with depression. There was a small effect size correlation between negative voice content and levels of worry. Use of defence behaviours (total, avoidance and in-situation) was correlated with negative voice content. There were medium effect size correlations for total defences and in-situation defences, and small effect size correlations for avoidance. There was a medium effect size correlation between positive voice content and well-being. No correlations (*r*<0.1) were found between voice content and meaningful activity, as measured on the time budget.

## Discussion

### Main findings and interpretation

This study provides a first insight into voice content regarding appearance. Strikingly, over 90% of the patients assessed reported experiencing voice content related to appearance and 50% were hearing negative content about their appearance on a daily basis. Both direct comments and attributions of others' evaluation of appearance were reported. Negative voice content most likely reflects the person's own concern about body image and self-esteem (i.e. there was consistency between voice comments and the person's own views of themselves). Voices commenting negatively on appearance was also associated with greater voice hearing severity, paranoia, depression and use of safety-seeking (defence) behaviours. This fits a clinical picture of negative voice content being associated with increased severity, distress and disruption. We suggest that those working psychologically with patients ask directly about the occurrence of such comments: it must be far from easy to hear voices regularly making personal comments about how you look.

Positive and negative voice content likely reflect self-perceived concerns regarding appearance and self-concept. In a recent qualitative study, patients with psychosis described how voice content about appearance reflected negative beliefs about the self: ‘The voices make comments on what I look like. They know all your fears, all your insecurities and they just play on it’.^6^ Patients described how these concerns led to ideas of reference and persecution, for example: ‘[the voices say] “You are ugly. You don't deserve nobody. You're ugly, people are looking at you, look at them looking at you”’.^6^ These findings suggest that concerns about appearance may be reflected in voice content and also in persecutory fears. In line with these patient accounts and evidence that voice content is often personally meaningful,^6,23^ our view is that hearing negative voices comment about how one looks may build upon pre-existing concerns about oneself and others. Subsequently this may exacerbate these fears and have a wide impact on day-to-day life. However, the current study provides no evidence of causality and this hypothesis must be further tested.

The current study provides the first indication that excess weight in particular is a common feature of appearance-related voice content in patients with psychosis. On a daily basis, negative comments regarding excess weight were the most frequently reported items. Perhaps this is unsurprising given three-quarters of the participants were overweight or obese, yet it has not been investigated before, despite the obvious potential consequences for such negative voices to impact on day-to-day life. Given the high rates of obesity,^1^ and severe physical health consequences among patients with psychosis^24^ this finding should not be overlooked. Longitudinal research is required to investigate the potential temporal and causal relationships between weight, appearance concerns and psychotic experiences such as negative appearance-related voice content. The early phase of treatment for psychosis, which is often associated with rapid weight gain^3^ represents a potentially important time for investigation and opportunity for prevention.

### Limitations

There are a number of limitations to the current study. The representativeness of the sample is a key issue. It is unknown how representative the current sample is of the wider population of patients with non-affective psychosis. Notably, patients were recruited on the basis of holding a current persecutory delusion, which, although a common presentation in services, is not the case for all patients hearing voices. Nor will this sample be representative of non-clinical voice hearers; content of voices has been identified as one feature that distinguishes those seeking help from those in the general population.^25^ Because of the novelty of the research question, there were no existing measures assessing voices and appearance. Therefore, a brief measure was developed. However, the measure has not been validated and beyond internal consistency the psychometric properties are currently unknown. Therefore, the measure requires further validation in a larger sample. Correlations are reported, but given the cross-sectional design of the study, it is not possible to determine causality or the potential impact of confounding variables. Different designs are now required to investigate this novel area.
